# Structures of PGAM5 Provide Insight into Active Site Plasticity and Multimeric Assembly

**DOI:** 10.1016/j.str.2017.05.020

**Published:** 2017-07-05

**Authors:** Apirat Chaikuad, Panagis Filippakopoulos, Sean R. Marcsisin, Sarah Picaud, Martin Schröder, Shiori Sekine, Hidenori Ichijo, John R. Engen, Kohsuke Takeda, Stefan Knapp

**Affiliations:** 1 Institute for Pharmaceutical Chemistry, Johann Wolfgang Goethe-University and Buchmann Institute for Molecular Life Sciences, Max-von-Laue-Strasse 9, 60438 Frankfurt am Main, Germany; 2 Nuffield Department of Clinical Medicine, Structural Genomics Consortium and Target Discovery Institute, University of Oxford, Old Road Campus Research Building, Roosevelt Drive, Oxford OX3 7DQ, UK; 3 Ludwig Institute for Cancer Research, University of Oxford, Old Road Campus Research Building, Roosevelt Drive, Oxford OX3 7DQ, UK; 4 Department of Chemistry and Chemical Biology, Northeastern University, Boston, MA 02115, USA; 5 Division of Cell Regulation, Graduate School of Biomedical Sciences, Nagasaki University, 1-14 Bunkyo-machi, Nagasaki 852-8521, Japan; 6 Laboratory of Cell Signaling, Graduate School of Pharmaceutical Sciences, The University of Tokyo, 7-3-1 Hongo, Bunkyo-ku, Tokyo 113-0033, Japan

**Keywords:** phosphoglycerate mutase, PGAM5, Ser/Thr phosphatase, catalysis, oligomerization, allosteric regulation, WDXNWD motif, histidine acid phosphatase, active site plasticity

## Abstract

PGAM5 is a mitochondrial membrane protein that functions as an atypical Ser/Thr phosphatase and is a regulator of oxidative stress response, necroptosis, and autophagy. Here we present several crystal structures of PGAM5 including the activating N-terminal regulatory sequences, providing a model for structural plasticity, dimerization of the catalytic domain, and the assembly into an enzymatically active dodecameric form. Oligomeric states observed in structures were supported by hydrogen exchange mass spectrometry, size-exclusion chromatography, and analytical ultracentrifugation experiments in solution. We report that the catalytically important N-terminal WDPNWD motif acts as a structural integrator assembling PGAM5 into a dodecamer, allosterically activating the phosphatase by promoting an ordering of the catalytic loop. Additionally the observed active site plasticity enabled visualization of essential conformational rearrangements of catalytic elements. The comprehensive biophysical characterization offers detailed structural models of this key mitochondrial phosphatase that has been associated with the development of diverse diseases.

## Highlights

• PGAM5 catalytic domain shares phosphoglycerate mutase fold and forms stable dimer
• WDPNWD motif allosterically activates the fully active dodecameric form
• Crystal structures reveal conformational plasticity of the PGAM5 active site

## Graphical Abstract

**Figure:**
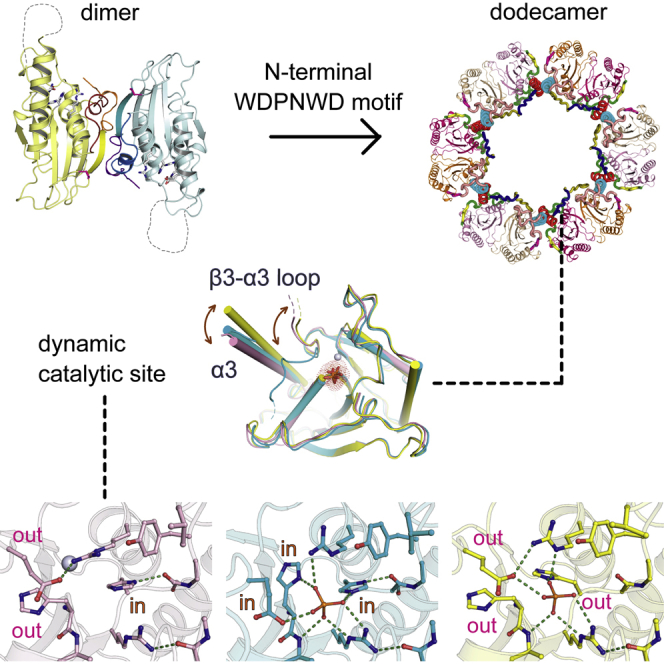

## Teaser

Chaikuad et al. present multiple crystal structures of PGAM5 and its biophysical analysis in solution, revealing the plasticity and structural mechanisms regulating oligomerization and its allosteric activation.

## Introduction

Phosphoglycerate mutase family member 5 (PGAM5) is a mitochondrial membrane protein that belongs to the phosphoglycerate mutase (PGAM) branch of the histidine acid phosphatase superfamily, known also as two-histidine phosphatase (2H-phosphatase). The PGAM catalytic domain shares homology with several metabolic enzymes, including PGAM1, PGAM2, 2,3-bisphosphoglycerate mutase (BPGM), and 6-phosphofructo-2-kinase/fructose-2,6-biphosphatase (PFKFB) (Jedrzejas, 2000, Rigden, 2008). Despite harboring a canonical RHGE catalytic motif, PGAM5 lacks a typical phosphotransferase and/or phosphohydrolase activity for small metabolites, and displays instead a phosphatase function specific for Ser/Thr and, potentially, histidine residues (Takeda et al., 2009, Panda et al., 2016). Hence, this protein is often referred to as a mitochondrial serine/threonine phosphatase, and classified as an atypical phosphatase alongside the closely related tyrosine phosphatases STS1 and STS2 (Lo and Hannink, 2006, Sadatomi et al., 2013).

PGAM5 was first discovered as a binding partner of the apoptosis regulator Bcl-X_L_ (Hammond et al., 2001), and subsequently as a substrate of the redox-regulated substrate adaptor KEAP1 that tethers the Cul3-dependent ubiquitin ligase complex of the KEAP1-Nrf2 protein degradation pathway at the outer membrane of mitochondria (Lo and Hannink, 2008). Several recent studies have further demonstrated that PGAM5 can associate with several complexes, and its phosphatase activity is essential for the regulation of various signaling pathways. For example, PGAM5 serves as an activator of ASK1 in MAPK pathways by dephosphorylating inhibitory sites present in this protein kinase, implicating a role in stress response (Takeda et al., 2009). In addition, PGAM5 is an essential regulatory component that promotes oxidative stress-induced necrosis/necroptosis in cancer cells through an association with the RIP1-RIP3-MLKL necrosome in mitochondria. This mitochondria attack complex initiates RIPK3 phosphorylation on PGAM5, which leads to the dephosphorylation and activation of DRP1, a guanosine triphosphatase that regulates an early and obligatory mitochondrial fission step for execution of programmed necroptosis (Lin et al., 2013, Lu et al., 2016, Moriwaki and Chan, 2013, Wang et al., 2012, Zhuang et al., 2013). Furthermore, several lines of evidence suggest that PGAM5 mediates apoptosis through interaction with Bcl-X_L_ promoting the degradation of this antiapoptotic protein, and through formation of the PGAM5-BAX-DRP1 complex (Wu et al., 2014, Xu et al., 2015, Zhuang et al., 2013). Nonetheless, its role in apoptosis and necroptosis has been subject to a debate in the recent literature (Ishida et al., 2012, Moriwaki et al., 2016, Remijsen et al., 2014).

Recently, a role of PGAM5 in mitophagy, a specialized autophagy program that antagonizes necroptosis by selectively degrading dysfunctional mitochondria to prevent an overproduction of reactive oxygen species, has been suggested (Liu et al., 2014, Lu et al., 2014, Lu et al., 2016, Wu et al., 2014). PGAM5 has been linked to two distinct mechanisms that regulate mitochondria homeostasis and mitophagic protection against cell necroptosis. First, it interacts and dephosphorylates the mitophagy receptor FUNDC1 (Chen et al., 2014, Wu et al., 2014) and second, it stabilizes PINK1, a kinase linked to early-onset Parkinson's disease. An impairment of this process in the PINK1-dependent mitophagy pathway through the loss of PGAM5 causes an accumulation of damaged mitochondria that worsen necroptosis, dopaminergic neuron degeneration, and defects in growth and survival, establishing a molecular link between PGAM5 and the pathogenesis of Parkinson's and cardiac diseases (Brenner et al., 2013, Imai et al., 2010, Kanamaru et al., 2012, Lu et al., 2014, Lu et al., 2016, Remijsen et al., 2014, Wai et al., 2016). Further investigation has linked PGAM5 to the regulation of immune responses and inflammatory diseases. In melanoma and acute inflammatory liver injury, RIPK3-activated PGAM5 regulates the dephosphorylation of DRP1 as well as NFAT to promote type 1 natural killer T cell activation and proinflammatory cytokine production, suggesting that the RIPK3-PGAM5-DRP1 signaling axis may mediate crosstalk between mitochondrial function and host immunity in these disease states (Kang et al., 2015).

Despite its important role in regulating important signaling processes, the molecular mechanisms regulating PGAM5 phosphatase activity remain largely unknown. To date, the only detailed biochemical characterization has revealed that PGAM5 has preference for phosphopeptide substrates containing negatively charged residues, and that phosphatase activity is allosterically controlled by an N-terminal extension containing the conserved WDXNWD motif that also regulates the interchangeable multimeric states (Wilkins et al., 2014). In this study, we determined crystal structures of the catalytic domain of human PGAM5, providing molecular models for its regulation and oligomeric assembly. In addition, the presented structures elucidated the structural role of the regulatory N-terminal WDXNWD motif in promoting the folding of the catalytic loop required for enzymatic activity and the formation of a dodecameric complex. In addition, the stability of the enzyme was probed in solution using hydrogen-exchange mass spectrometry. The structural insights at various phosphate-binding states may potentially reflect the conformational changes during catalysis that may be also relevant for other members of the 2H-phosphatase family.

## Results and Discussion

### Overall Structure of PGAM5 Catalytic Domain

**Figure 1:**
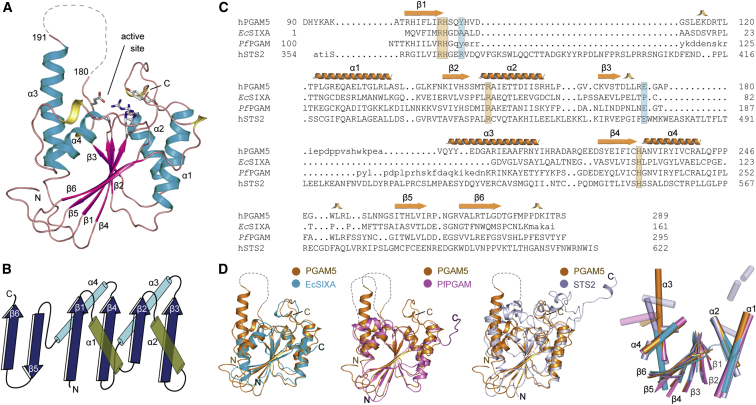
Overall Structure of PGAM5 and Comparison with Other PGM Family Members (A) Overall structure of PGAM5. The main secondary structure elements are labeled. The disordered loop region following helix α3 is indicated by a dotted line. (B) Schematic representation of the PGAM5 secondary structure arrangement. (C) Structure-based sequence alignment of PGM family members. Secondary structural elements shown at the top of the alignment are from the PGAM5 structure (see Figure S1 for extended alignment). (D) Detailed comparison of the catalytic core fold of three PGM enzymes (colored as indicated in the figure) with PGAM5 (orange) as well as arrangement of the core secondary structure elements (right panel).

Among a series of truncated proteins, the ΔN90-PGAM5 construct harboring the catalytic domain readily crystallized, enabling high-resolution structure determination to 1.70 Å (Table 1). Overall, the catalytic domain of PGAM5 adopted a classical phosphoglycerate mutase-like fold with the canonical three-layer sandwich topology, which featured a six-stranded β sheet flanked on both sides by α helices with the first four parallel strands arranged in an alternated β/α manner (Figures 1A and 1B). The last two strands formed an antiparallel arrangement, projecting the C-terminal tail in proximity to the active site situated on top of the β-sheet core. Such “fold-back” topology of the C-terminal tail has been regarded as a common characteristic among members of the PGM superfamily, with a potential role in enzyme activity and substrate specificity (Rigden, 2008, Walter et al., 1999). The β3-α3 loop region situated in proximity to the active site (residues 180–190) was disordered and therefore was excluded from the final model.

**Table 1:** Data Collection and Refinement Statistics

	PGAM5 ΔN90 Iodide Derivative	PGAM5 ΔN90 Apo	PGAM5 ΔN90 + Phosphate	PGAM5 ΔN54 + Phosphate
PDB ID		3MXO	3O0T	5MUF

**Data Collection**				

Beamline	Rigku FR-E	Rigku FR-E	Rigaku FR-E	Diamond Light Source, i03
Wavelength (Å)	1.54178	1.54178	1.54178	0.97630
Resolution^a^ (Å)	40.67–2.25 (2.37–2.25)	29.48–1.70 (1.79–1.70)	36.54–1.90 (2.00–1.90)	41.03–3.10 (3.27–3.10)
Spacegroup	*P*2_1_2_1_2_1_	*P*2_1_2_1_2_1_	*P*2_1_2_1_2_1_	*I*222
Cell dimensions	*a* = 71.4, *b* = 71.7, *c* = 81.4 Å	*a* = 70.7, *b* = 70.9, *c* = 81.5 Å	*a* = 71.0, *b* = 73.1, *c* = 81.3 Å	*a* = 82.1, *b* = 141.0, *c* = 182.9 Å
	α = β = γ = 90.0°	α = β = γ = 90.0°	α = β = γ = 90.0°	α = β = γ = 90.0°
No. of unique reflections^a^	20,446 (2,896)	45,512 (6,408)	34,056 (4,896)	18,243 (2,709)
Completeness^a^ (%)	100.0 (100.0)	99.5 (97.6)	100.0 (100.0)	94.3 (96.2)
*I*/σ*I*^a^	15.1 (3.0)	16.7 (2.2)	15.8 (2.2)	6.8 (2.0)
*R*_merge_^a^	0.077 (0.542)	0.053 (0.644)	0.049 (0.669)	0.271 (0.993)
*R*_pim_^a^	0.041 (0.263)	0.026 (0.325)	0.025 (0.347)	0.101 (0.373)
Redundancy^a^	5.9 (5.9)	4.8 (4.6)	4.7 (4.6)	7.0 (6.9)

**Refinement**				

No. of atoms in refinement (P/L/O)^b^		3,137/–/410	3,086/10/236	5,062/15/51
*R*_fact_ (%)		16.8	18.3	22.7
*R*_free_ (%)		20.3	22.8	25.6
B factor (P/L/O)^b^ (Å^2^)		26/–/39	44/38/49	51/38/22
RMSD bond^c^ (Å)		0.016	0.016	0.010
RMSD angle^c^ (°)		1.5	1.7	1.1

**MolProbity Ramachandran**				

Favored (%)		99.19	98.40	94.98
Outlier (%)		0	0.27	0

a Values in parentheses show the statistics for the highest-resolution shells.

b P/L/O indicate protein, phosphate molecules presented in the active sites, and other (water and solvent molecules), respectively.

c RMSD, root-mean-square deviation.

### Similarity of PGAM5 to Other PGM Family Members

The structural similarity search using DALI revealed that PGAM5 was highly homologous to a variety of enzymes with diverse functions from the PGM/AcP superfamily (maximum *Z* score of ∼20), despite sharing sequence identities of less than 20% with an exception of ∼40% for *Plasmodium falciparum* PGAM2 (*Pf*PGM2). To further evaluate this result, we next performed structural comparison with three phosphatases in this family, including the closely related phosphatase *Pf*PGM2 (Hills et al., 2011), the histidine acid phosphatase archetype *Escherichia coli* SixA (*Ec*SixA) (Hamada et al., 2005), and the PGM domain of human STS2 that exhibits tyrosine phosphatase activity (Chen et al., 2009). In agreement with the DALI results, the core architectures of all four proteins superimposed well with all catalytically important residues located at similar positions (Figures 1C, 1D, and S1).

Nonetheless, structural alterations were observed for the β1-α1 loop, the α3 helix, and the C-terminal tail. The former loop of PGAM5 had similar length and conformation to that of *Ec*SIXA and *Pf*PGM2, but was much shorter than the one present in STS2 which harbored a large insertion. The helix α3, including the preceding loop, was one of the most divergent regions. Not only was the helix in PGAM5 much more extended (28 Å compared with 20, 11, and 23 Å in *Ec*SIXA, *Pf*PGM2, and STS2, respectively), its vertical trajectory was tilted away from the core and was unique when compared with the compact arrangement of the core in related PGM structures. Furthermore, a diverse structural arrangement was noted for the C-terminal tail, where the fold-back conformation in PGAM5 differed significantly from the conformations observed in the other family members, such as the partially disordered C terminus in *Ec*SIXA, exterior outward positioning of the C-terminal tail in *Pf*PGM2, and the tail exchange with a neighboring active site within the dimer in STS2. In addition, the high sequence variation within these regions was also noted, particularly at the β3-α3 loop region and around Y108 and E177 in PGAM5, which contributed toward the formation of the active site (see below). Interestingly, these sequence variations were located in close proximity to the catalytic pockets, implying that despite the evolutionarily conserved core architecture, variations within these regions could be central to diverse functions of PGMs and their substrate specificity.

### Dimeric Assembly of PGAM5

**Figure 2:**
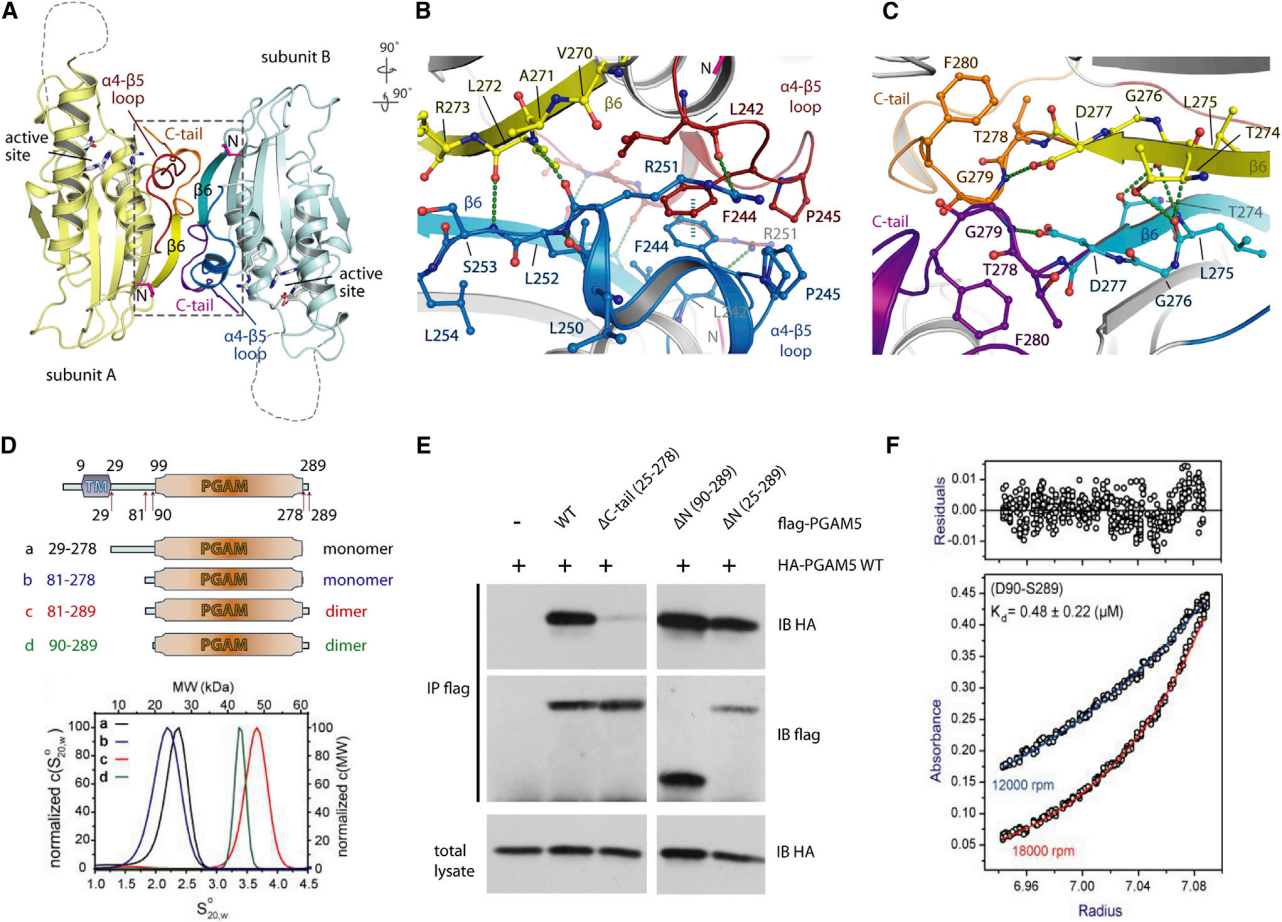
Association of the PGAM5 Dimer (A) Ribbon diagram showing the dimer interface. The position of the active site as well as the interacting secondary structure elements are labeled. (B) Details of the dimer interface between β6 and the C-terminal tail. Secondary structure elements are colored in the same way as shown in (A). (C) Details of the dimer interface in the β-sheet region. (D) Schematic of the constructs studied (upper panel) and AUC sedimentation velocity data (lower panel). Shown is a normalized distribution of sedimentation coefficients (S_20, w_) and the derived molecular weights determined from sedimentation velocity experiments. (E) Immunoprecipitation experiment using HA-tagged full-length protein and FLAG-tagged truncated constructs. IP, immunoprecipitation; IB, immunoblotting; WT, wild-type. (F) AUC sedimentation equilibrium experiment of construct “d” (residues 90–289) measured at two velocities. Residuals of a non-linear least-squares fit to a dimer association model are shown in the upper panel.

The two molecules of PGAM5 in the crystallographic asymmetric unit demonstrated a dimeric arrangement. Both subunits associated by aligning the β-sheet cores along the non-crystallographic two-fold symmetry with their active sites located at distal sites (Figure 2A). Intermolecular interactions were exclusively constrained to the C-terminal portions, where the α4-β5 loop, β6 strand, and C-terminal tail from one molecule packed against the corresponding region in the other protomer. The α4-β5 loop from one protomer created a number of interactions to its analogous region in the interacting dimer molecule including the N terminus of β6 strand. Interactions comprised π-stacking of the F244, R251-L242 carbonyl backbone, and several backbone hydrogen bonds in the β6 strand (Figure 2B). The β6 strands from both subunits interacted with a short stretch of residues (274–277) by formation of a network of hydrogen bonds (Figure 2C). Thus, the dimeric assembly of the β strands created a single β-sheet structure in this quaternary arrangement.

### The C-Terminal Tail Is Important for Dimer Formation

Unlike the α4-β5 loop and β6 strand, the contribution of the C-terminal tails to dimeric intersubunit contacts was limited to only one hydrogen bond between the β6 D277 and the tail residue G279 (Figure 2C), prompting us to investigate the role of this region regarding the stability of the dimer. To address this question, we designed and purified four constructs of PGAM5 with various N- and C-terminal truncations and studied self-association using analytical ultracentrifugation (AUC) (Figure 2D). As expected from our structural data, the two constructs encompassing the intact C-terminal tails (residues 81–289 and 90–289) migrated as ∼48- or 43-kDa species, confirming a dimeric state. In contrast, the other two constructs lacking the C-terminal tail (residues 29–278 and 81–278) exhibited much lower sedimentation coefficients of ∼2.2–2.4 Svedberg units, suggesting the predominant monomeric forms in solution. We next performed immunoprecipitation pull-down assays using various FLAG-tagged truncated constructs and the hemagglutinin (HA)-tagged full-length PGAM5 (wild-type). Consistently, the deletion of the C-terminal tail (ΔC-tail) almost completely abolished the association of this construct with full-length protein, an ability that was highly maintained in the other truncated variants retaining the C-terminal tail (Figure 2E). These results therefore suggested that the C-terminal tail played an important stabilizing role in the dimeric assembly, which was also supported by the dissociation constant (K_D_) of ∼0.48 μM determined for the construct “d” (residues 90–289) by AUC equilibrium sedimentation experiments (Figure 2F).

### Structure of ΔN54-PGAM5 Harboring the Conserved WDXNWD N-Terminal Motif

**Figure 3:**
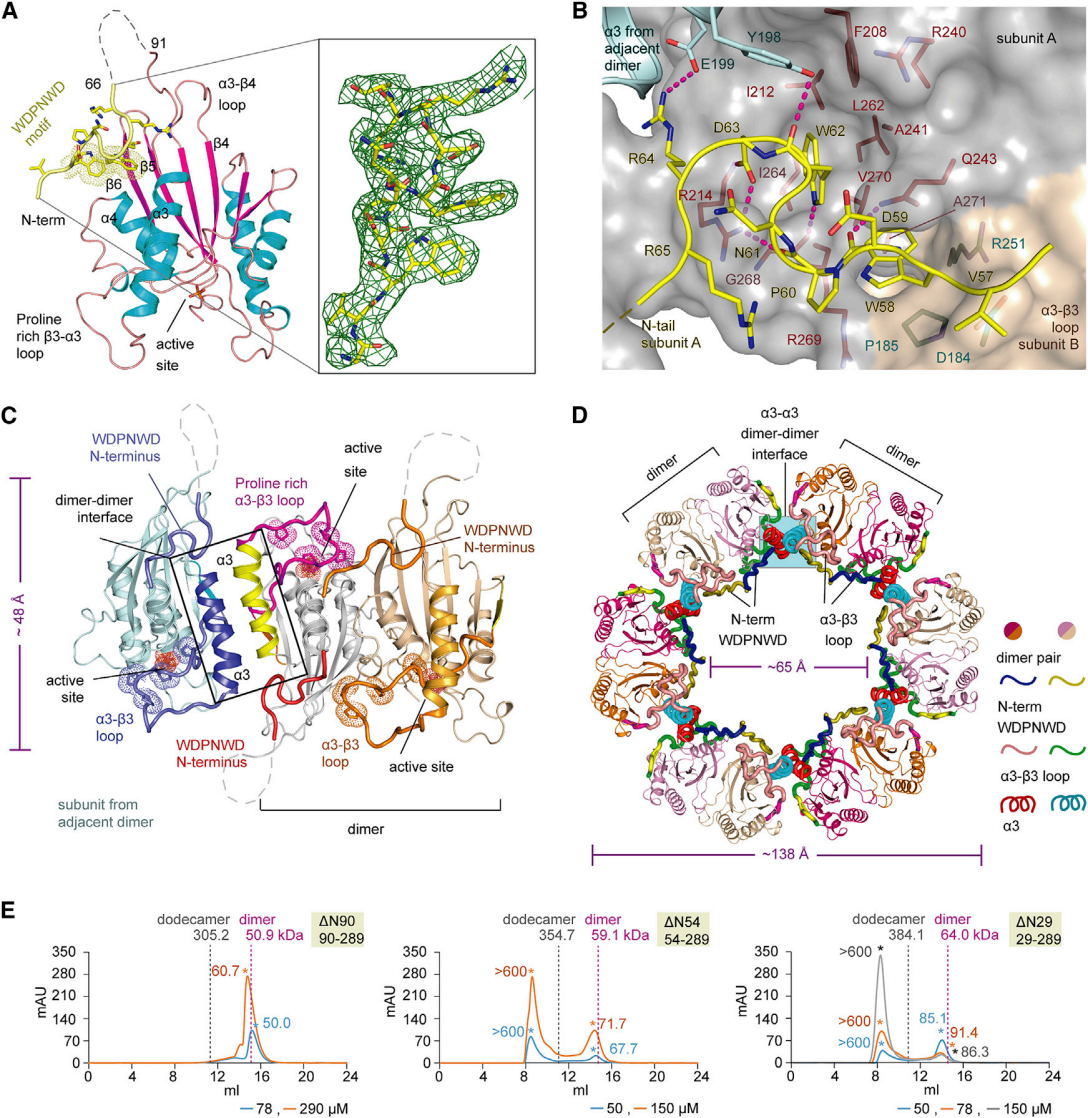
Structure of PGAM5 Containing the N-terminal WDPNWD Motif (A) Secondary structure of the N-terminally extended construct. The electron density around the WDPNWD motif (|2F_o_| − |F_c_| omitted map contoured at 1σ) is shown. (B) Details of the interaction of the WDPNWD motif. Shown is the WDPNWD motif (stick representation) and molecular surfaces contributed by various PGAM5 subunits in the dodecameric assembly. Hydrogen bonds are shown as dotted lines. (C) Arrangement of two PGAM5 dimers within the asymmetric unit. The main structural elements are labeled and the location of the WDPNWD motif is indicated. (D) Dodecameric arrangement of PGAM5 constructed by applying crystallographic symmetry. Interfaces and structural elements important for dimer and oligomer formation are highlighted as shown in the figure capture. (E) Size-exclusion chromatograms of three PGAM5 constructs. Asterisks indicate calculated molecular mass of the peak, and dashed lines indicate expected elution points for dimer and dodecamer based on standard protein markers (see Figure S2).

Previous studies have demonstrated that the N-terminal region harboring the conserved WDXNWD motif is essential for both full phosphatase activity and multimeric assembly of PGAM5 (Wilkins et al., 2014). To provide structural insights into the role of the N terminus, we next determined the structure of the longer ΔN54-PGAM5 in complex with a phosphate ion. In all three molecules in the asymmetric unit, although most parts of the N-terminal extension to the catalytic domain (amino acids 67–90) was disordered, clear electron density allowed a confident assignment of the WDPNWD motif within the N-terminal region, which packed along the surface of the catalytic core (Figure 3A). The accommodation of the WDPNWD motif required both intra- and interdimeric contacts where the tryptophan residues of this motif were accommodated in two hydrophobic grooves, located at intermolecular interfaces created by two different PGAM5 subunits (Figure 3B). The first pocket accommodating W58 was formed at the dimer interface created by β6, α4, and the neighboring β3-α3 loop, while the other groove forming the interaction site for W62 was situated between two dimers created largely by the α3, α4, β5, and β6 with contributions of Y198 and E199 located in the α3 from a subunit of an adjacent dimer.

### The Role of the WDXNWD Motif in Allosteric Regulation and Dodecameric Assembly

The presence of the WDPNWD motif triggered a simultaneous occurrence of two interesting structural features that were not observed in the short ΔN90-PGAM5 structures, including the now ordered proline-rich β3-α3 loop and the newly formed interdimeric interactions resulting in a dodecameric assembly. Within the asymmetric unit, one subunit of the dimer associated with the subunit of an adjacent dimer with their α3 helices paired in a reverse manner at the dimer-dimer interface (Figure 3C). The WDPNWD-containing N terminus was then positioned in proximity to the proline-rich β3-α3 catalytic loop, leading to an alternated arrangement that resulted in the β3-α3 catalytic loop of one molecule being sandwiched between two neighboring N termini, one from the subunit within the dimer and the other from the protomer of the adjacent dimer. This configuration promoted most likely an ordering of the loop that in turn completed the active site architecture, a feature not observed in the ΔN90-PGAM5 structure (Figure 3C). This in *trans* effect of two neighboring WDPNWD-containing N termini toward the ordering of the catalytic loop was consistent with the previous experiment showing an activation of the PGAM5 catalytic domain by a WDPNWD-containing peptide (Wilkins et al., 2014), and therefore provided structural evidence for the allosteric regulatory role of the WDPNWD motif previously proposed (Wilkins et al., 2014).

Another consequence was observed in the formation of a large assembly in the crystals. Based on the dimer-dimer arrangement, an application of the crystallographic symmetry led to a single-layer, doughnut-like dodecameric assembly with an outer dimension of 138 × 48 Å and a radius of 65 Å for the inner hole (Figure 3D). This large dodecamer was in excellent agreement with the previously suggested quaternary structure in solution (Wilkins et al., 2014). To further confirm the existence of such a large oligomer in solution, we performed size-exclusion chromatography (SEC) on three truncated proteins including ΔN90-, ΔN54-, and ΔN29-PGAM5, the latter of which was the closest form of the protein present in cells through proteolytic cleavage by PARL (Figures 3E and S2). As expected, the catalytic domain alone (ΔN90) lacked an ability to form large assemblies, and existed as a dimer in solution even at high protein concentration. The elution patterns of the other two forms (ΔN54 and ΔN29) were remarkably similar, including a population of the dimeric form that decreases with increasing protein concentration. Surprisingly, the large species did not elute at a retention volume expected for a dodecamer. However, the asymmetry of the disk-like dodecameric structure explains the unusual retention. The large size of the disk radius (138 Å) probably led to complete exclusion of the PGAM5 oligomer from SEC beads (Figure S2). Although the oligomeric state should be confirmed by alternative experimental techniques such as small-angle X-ray scattering in solution, we find it conceivable that the dimeric arrangement of the catalytic domain functions as a primitive building block for the formation of a large, most likely dodecameric assembly in ΔN54-PGAM5 and ΔN29-PGAM5 observed in the crystal structure. Nonetheless, since our data were based on the recombinant proteins, further experiments would be required to confirm the oligomeric states of full-length PGAM5, which may exist in multiple oligomeric states in cells (Wilkins et al., 2014).

### The WDXNWD Motif Confers Stability of PGAM5

**Figure 4:**
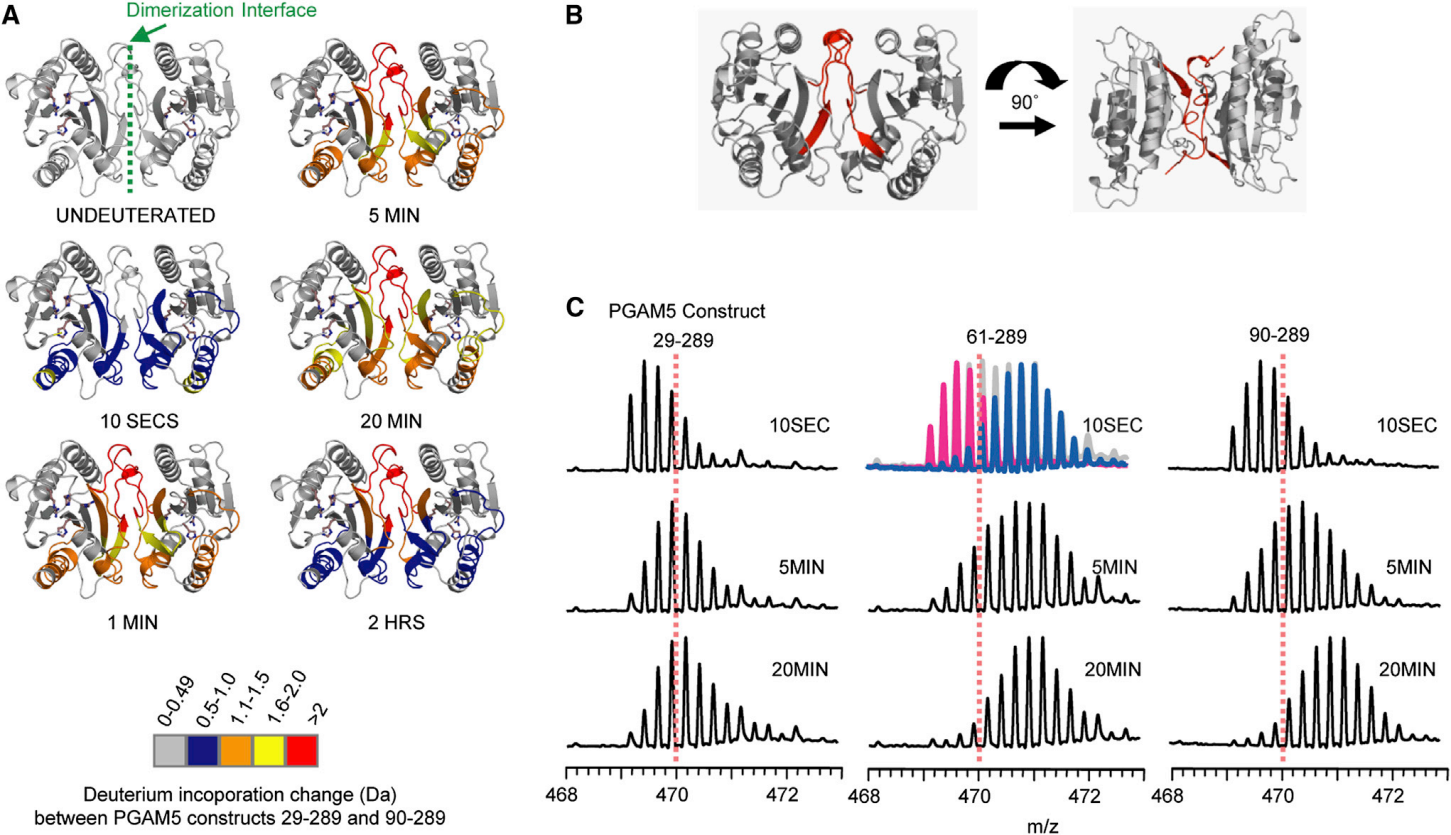
HXMS Analysis of PGAM5 Constructs (A) Deuterium incorporation differences between the two most extreme N-terminal truncation constructs ΔN29-PGAM5 and ΔN90-PGAM5 mapped onto the dimer structure of ΔN90-PGAM5 for the various determination time points. The dimerization interface is indicated by the green dashed line in the panel depicting the undeuterated protein. (B) The peptic peptide encompassing the C-terminal tail (residues 270–289) located at the dimerization interface with measurable EX1 deuterium incorporation is shown in red. (C) Deuterium incorporation mass spectra for the peptic peptide encompassing the C-terminal tail residues 270–289 for PGAM5 constructs ΔN29-PGAM5, ΔN61-PGAM5, and ΔN90-PGAM5. The dashed lines affixed at *m*/*z* 470 are provided as a visual guide to assess deuterium incorporation. The presence of EX1 kinetics in the ΔN61-PGAM5 peptic peptide is highlighted in the 10-s time point (middle panel) as pink and blue conformational populations.

To assess whether the structural alterations induced by the WDPNWD motif observed in the crystal structure play a role in protein stability, we next performed hydrogen-exchange mass spectrometry experiments. The experiments revealed remarkably different deuterium incorporation patterns among various PGAM5 constructs with different N-terminal truncations in agreement with the different oligomeric states observed. The patterns were similar for the enzymatically active constructs (ΔN29-PGAM5 and ΔN54-PGAM5) while the less active forms (ΔN61-PGAM5 and ΔN90-PGAM5) incorporated deuterium more rapidly (Figure 4A). The location of differences in deuterium incorporation was centralized around two regions, including the β3-α3 loop catalytic loop (residues 175–209), which was disordered in the ΔN90-PGAM5 structure but was well ordered in the N-terminally extended structure comprising the WDPNWD motif, and the C-terminal tail contributing to dimerization (residues 270–289). The ΔN61-PGAM5 protein lacking a part of the WDXNWD motif displayed unique EX1 deuterium incorporation patterns in the dimerization interface indicative of multiple conformational states in solution. For comparison, since both active ΔN29-PGAM5 and ΔN54-PGAM5 shared the same behavior with regard to deuterium incorporation, we next focused on the EX1 deuterium incorporation patterns for the C-terminal tail peptic peptide (residues 270–289) located at the dimeric interface of the three selected constructs ΔN29-PGAM5, ΔN61-PGAM5, and ΔN90-PGAM5 (Figure 4B). As expected, the deuterium incorporation pattern for this peptic peptide for ΔN29-PGAM5 was different to that of ΔN61-PGAM5 and ΔN90-PGAM5 (Figure 4C). We observed that the EX1 deuterium incorporation pattern was completely abolished by the presence of the intact WDXNWD motif in ΔN29-PGAM5, indicating that these residues likely associate with the PGAM5 core structure. Therefore, our results suggested that the presence of the WDPNWD motif led to different conformations of the protein and appeared to enhance the stability of the enzyme, which in addition to the roles in allosteric regulation and large multimeric formation could be essential for the proper function of PGAM5's phosphatase activity as proposed previously (Wilkins et al., 2014).

### PGAM5 Shares a Conserved PGM Catalytic Center

**Figure 5:**
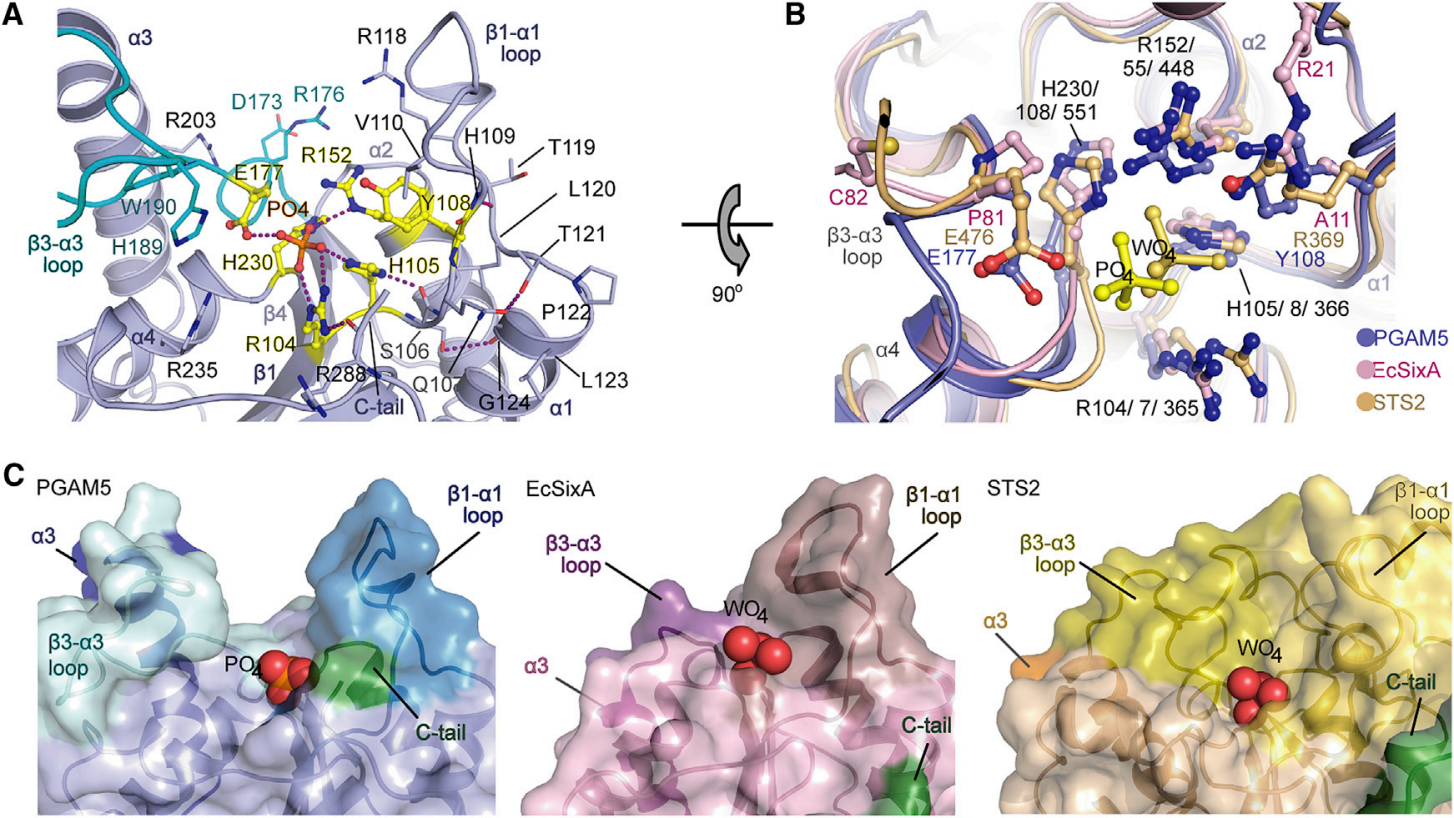
Active Site Comparison of PGM Family Members with PGAM5 (A) Coordination of the co-crystallized phosphate ion in ΔN54-PGAM5. The main active site residues are labeled. Hydrogen bonds are shown as dotted lines. (B) Rotated view of the active site and comparison with the actives sites of *Ec*SixA and STS2. Main-chain traces and carbon atoms are colored by protein structure as indicated in the figure. (C) Surface representations of the active sites of PGAM5, *Ec*SixA, and STS2. The co-crystallized phosphate ion or the phosphate mimetic WO_4_ ions are highlighted as solid spheres.

The ΔN54-PGAM5-phosphate complex allowed us to investigate the fully ordered state of the PGAM5 active site. The phosphate molecules interacted with the catalytic center located on top of the β-sheet core structure and were coordinated by loop regions linking β1, β4, and α4 (Figure 5A). The active site was lined by the invariant positively charged catalytic cluster including the canonical two histidine residues H105 and H230, in addition to the arginine residues R104 and R152, which adopted the 2H-phosphatase signature arrangement required for PGM catalytic activities. The arginine-histidine pair R104 and H105 constitutes the characteristic RHG motif, which harbors a conserved sequence variation to RHS in PGAM5. This substitution did not perturb the integrity of the fold, as the small polar serine side chain did still fit within the α1 hydrophobic groove created by the conserved L(S/T)XXG motif (_120_LTPLG_124_ in PGAM5). The serine formed an additional hydrogen bond to the Gly124 carbonyl backbone. Completion of the active site was achieved by an additional two residues, Y108 C-terminal of the RHS motif and E177 located at the N terminus of the β3-α3 loop, situated at either side of the active site pocket (Figure 5A).

Structural comparison with other members revealed that despite the conservation of the histidine and arginine catalytic signature motif, some distinct features were observed in the PGAM5 active site. These included sequence variations at positions occupied by Y108 and E177 (Figure 5B). For the former, the presence of a bulky tyrosine residue at this position was rather unique in comparison with the typical arginine residue, commonly found in both branch-2 AcPs and small numbers of branch-1 enzymes, such as STS1/2, or a much smaller residue, such as glycine, alanine, serine, or threonine, found mainly in a majority of branch-1 PGM enzymes including *Ec*SIXA (San Luis et al., 2013). It was observed that the presence of small amino acids at this position in the branch-1 PGMs was often compensated by an adjacent positively charged or polar residue, such as R25 in *Ec*SIXA, of which the protruding side chain was observed to overlap with the PGAM5 tyrosine Y108 and corresponding arginine residues in other family members (Figure 5B). The presence of a polar or positively charged residue at this position is most likely functionally essential, as previously demonstrated in STS1/2 (San Luis et al., 2013). Furthermore, another significant difference was noted for the deep active site cleft and the narrow shape of the substrate-binding site, which was fenced primarily by the vertically extended α3 and the preceding β3-α3 loop. This confined pocket with a width of only ∼14 Å was markedly different to the open and flat active sites of the other PGM family members such as STS2 and *Ec*SIXA (Figure 5C). Interestingly, this cleft was lined by a number of positively charged residues (Figure 5A), supporting the preference of PGAM5 for acidic peptide substrates (Wilkins et al., 2014). Overall, these distinct features of the PGAM5 active site provide a structural rationale for its distinct substrate specificity.

### Conformational Plasticity of the PGAM5 Catalytic Site

**Figure 6:**
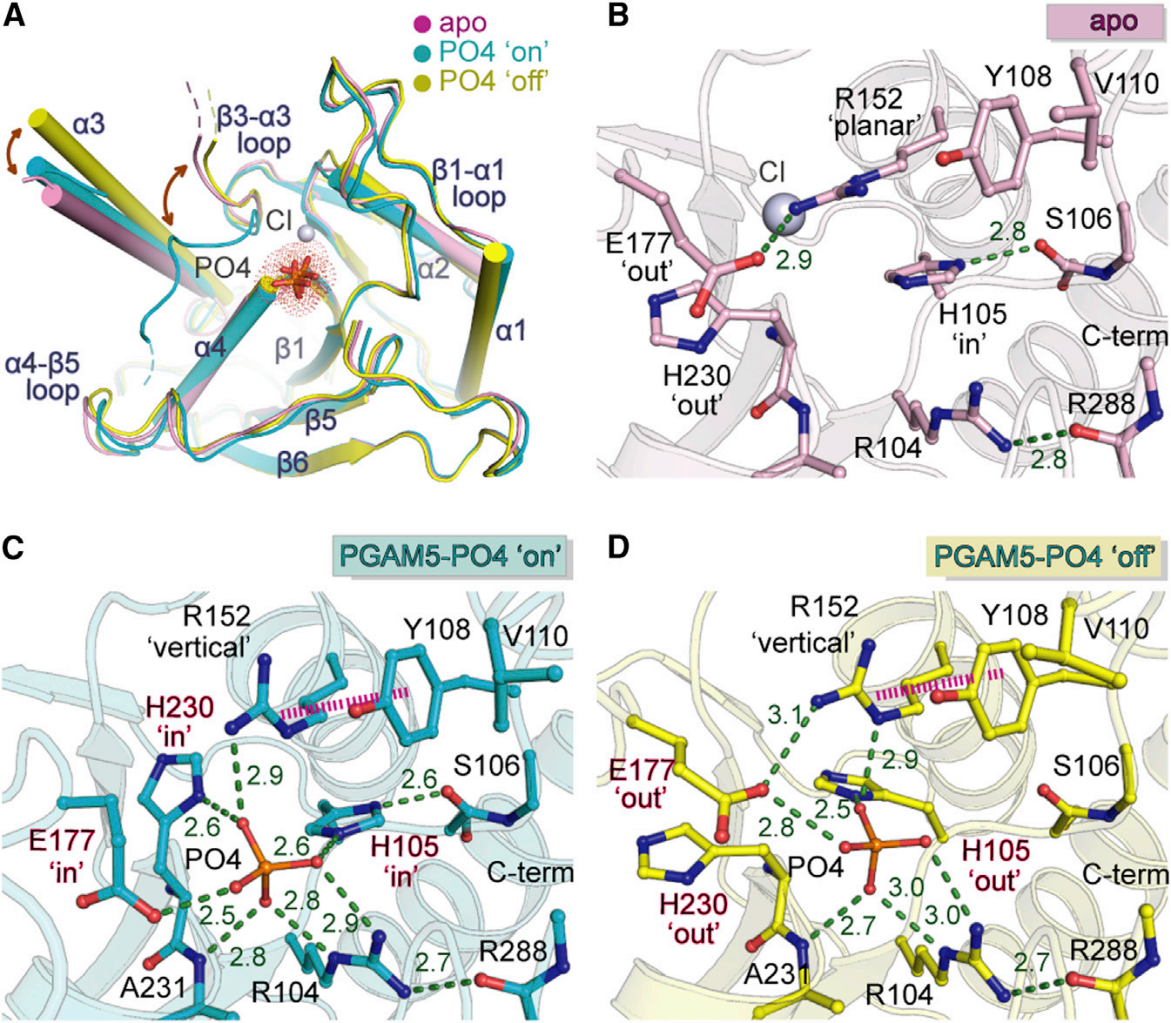
Plasticity of the PGAM5 Active Site (A) Structural comparison of three active site conformations comprising the apo enzyme and two phosphate-bound states termed “on” and “off.” The bound phosphate ions are shown in stick representation, (B) Details of side-chain conformations of catalytically important residues in the apo-PGAM5 active site. (C) Structural details of the phosphate interaction in the catalytically competent “on” state. (D) Details of the inactive “off” phosphate-bound state preparing the enzyme for product release and conversion to the unligated apo state.

To provide high-resolution details of the catalytic center, we also determined the high-resolution structure of ΔN90-PGAM5 in complex with phosphate (Table 1). Surprisingly, comparative structural analyses revealed that while the overall structures remained remarkably similar, subunits in both the dimeric ΔN90-PGAM5-phosphate complex and the apo structure exhibited a number of significant differences primarily within the β3-α3 loop, the α3 helix, and the side chains of the catalytic residues, leading to three distinct conformations of the enzyme that we classified as (1) a phosphate-free form (apo), (2) a phosphate-bound “on” state (on), and (3) a phosphate-bound “off” state (off) (Figure 6).

The β3-α3 loop was the region that showed the highest degree of flexibility with two major conformations observed, an opened form in the apo and off states and a closed form in the on state (root-mean-square deviation [RMSD] of ∼0.9 Å with an average Cα shift of ∼3.9 Å for residues 175–179) (Figure 6A). Open-to-closed molecular switch moved the β3-α3 loop toward the phosphate-binding pocket, during which the residues at the tip of the loop underwent a positional shift of ∼8 Å. Due to their close proximity, the movement of the loop might occur concurrently with the rearrangement of the neighboring α3 helix, of which a swing movement of up to 14° was apparent. In addition, structural alterations of the catalytic residues, including the four conserved histidine and arginine, Y108 and E177, were also evident, resulting in slight changes in both orientations and binding modes of phosphate groups in the different states of the enzyme. Overall, the conformational rearrangements of the active site between three different phosphate-bound states demonstrated the plasticity of PGAM5, which could be functionally essential for the phosphatase activity as described in the section below.

### Molecular Switch of Catalytic Residues between Apo, “On,” and “Off” States

Close inspections of the catalytic center further revealed differences in the conformations of the catalytic residues between the apo, “on,” and “off” states of the PGAM5 dimer (Figures 6B–6D). In the phosphate-free apo state, the nucleophile H105 adopted an inward conformation, of which the imidazole ring formed a hydrogen bond contact to the S106 carbonyl backbone and was sandwiched between the guanidinium groups of R104 and R152 that oriented perpendicular and co-planar to H105, respectively. In addition, an open conformation of the β3-α3 loop kept E177 in an outward-oriented conformation, allowing an outward swing of the H230 side chain.

Binding of a phosphate ion induced a number of rearrangements in the active site resulting in two distinct on and off states, which were termed in relation to the “in” and “out” conformation of H105, respectively (Figures 6C and 6D). In the on state, in addition to H105 all catalytic residues were observed to assume a potentially catalytically active position (Figure 6C). This conformation was most likely induced by the closure of the β3-α3 loop which moved E177 inward, triggering an inward swing of H230. In a concerted manner, the “in” H230 likely forced an approximately 90° rotation of R152 side chain orienting perpendicular to H105 and planar to Y108 for cation-π stacking. This rearrangement resulted in a compact active site, enabling a number of contacts between the protein and the bound phosphate. In addition, it is interesting to note that not only H105 was sterically locked in an “in” conformation, the “in” movement of E177 was particularly interesting due to its proposed role as a general base for the activation of a nucleophilic water (Chen et al., 2009).

In comparison, the stark difference of the off state of the enzyme was characterized by an “out” position of H105, rotating away from its catalytically competent position. This movement was likely a consequence of steric constraints created by R104 and R152 that retained their “vertical” conformations (Figure 6D). This conformation was stabilized by the open β3-α3 loop, which moved E177 into an “out” position allowing also an outward swing of H230. Although this conformation maintained affinity for phosphate, the all-out rearrangement of the catalytic residues presumably depicted a catalytically incompetent state of the enzyme.

Overall, the unseen conformational alteration of the PGAM5 catalytic elements suggested a high degree of plasticity of the catalytic center, which might depict conformational rearrangements essential for the dephosphorylation reaction. Due to highly conserved catalytic machinery, the structural insights presented here may also help to elucidate the structural mechanism that regulates catalysis across the histidine acid phosphatase superfamily.

## STAR★Methods

### Key Resources Table

**Table:** 

REAGENT or RESOURCE	SOURCE	IDENTIFIER
**Antibodies**		

Anti-flag M2 affinity gel	Sigma	Cat# A2220, RRID: AB_10063035
Anti-HA high affinity, clone 3F10	Sigma	Cat#ROAHAHA

**Bacterial and Virus Strains**		

*Escherichia coli* Rosetta	Novagen	Cat#70954

**Chemicals, Peptides, and Recombinant Proteins**		

PEG smears	Molecular dimension	www.moleculardimensions.com
PEG 3350	Molecular dimension	Cat#PEG 3350
PEG 5000 MME	Molecular dimension	Cat#PEG 5000 MME
HEPES	Fisher	Cat#BP310
Tris-base	Fisher	Cat#BP152
MES	Molecular dimension	Cat#MD2-013

**Deposited Data**		

PGAM5 ΔN90, apo	This study	PDB: 3MXO
PGAM5 ΔN90, phosphate complex	This study	PDB: 3O0T
PGAM5 ΔN54, phosphate complex	This study	PDB: 5MUF

**Experimental Models: Cell Lines**		

HEK293	Sigma	Cat# 85120602

**Recombinant DNA**		

pNIC28-Bsa4 plasmids encoding PGAM5 gene	This study	N/A
pcDNA3 plasmids encoding PGAM5 gene	This study	N/A

**Software and Algorithms**		

MOSFLM	Powell et al., 2013	http://www.ccp4.ac.uk
SCALA	Evans, 2011	http://www.ccp4.ac.uk
SHELXD	Sheldrick, 2010	http://www.ccp4.ac.uk
SHARP	Bricogne et al., 2003	https://www.globalphasing.com/sharp/
Buccaneer	Cowtan, 2012	http://www.ccp4.ac.uk
PHASER	McCoy et al., 2007	http://www.ccp4.ac.uk
COOT	Debreczeni and Emsley, 2012	http://www.ccp4.ac.uk
REFMAC	Rinaldelli et al., 2014	http://www.ccp4.ac.uk
MOLPROBITY	Hintze et al., 2016	http://molprobity.biochem.duke.edu/
SEDFIT	Schuck, 2000	http://www.analyticalultracentrifugation.com
Ultraspin	MRC Cambridge	http://www.biophysics.bioc.cam.ac.uk

**Other**		

Superdex Increase s200 10/300 GL	GE healthcare	Cat# 28990944
nanoACQUITY UPLC system with HDX technology	Waters	http://www.waters.com
ECL western blotting system	GE healthcare	Cat# RPN2108

### Contact for Reagent and Resource Sharing

Further information and requests for reagents should be directed to the lead contact, Apirat Chaikuad (chaikuad@pharmchem.uni-frankfurt.de).

### Method Details

#### Protein Purification

All PGAM5 constructs were subcloned into pNIC28-Bsa4 incorporating an N-terminal TEV-cleavable His_6_ tag. The recombinant proteins were expressed in *E. coli* Rosetta strain cultured in LB media at 37°C to OD_600_ of 0.6-0.8 before transferring to 18°C for an induction overnight with 0.5 mM IPTG. Cells were harvest and re-suspended in 50 mM HEPES, pH 7.5, 500 mM NaCl, 5 mM imidazole, 5% glycerol and 0.5 mM TCEP. After lysis by sonication, the supernatant was separated by centrifugation. The recombinant protein was initially purified using Ni^2+^-affinity chromatography, followed by TEV cleavage overnight at 4°C. The histidine tag and TEV protease were removed by passing the cleaved protein through nickle sepharose resin. Subsequent size exclusion chromatography was performed in buffer containing 50 mM Tris, pH 7.5, 300 mM NaCl and 0.5 mM TCEP.

#### Crystallization

Recombinant ΔN90-PGAM5 and ΔN54-PGAM5 were concentrated to 10-15 mg/mL, and used for sitting drop crystallization at 20°C. For ΔN90-PGAM5, initial hits were identified in the PEG smears-based screen with various conditions containing PEG smears and buffer pH 6-8 shown to promote the crystals (Chaikuad et al., 2015). Deconvolution of PEG smears identified the effective conditions, containing the mixture of PEG 3350 and 5000 MME at 12-20% and 0.1 M HEPES, pH 7.5, which were used for growing both apo and the phosphate complex. For experimental phasing, the apo-crystals were soaked with 0.5 M potassium iodide for 10 minutes. Crystallization of the ΔN54-PGAM5-phosphate complex was performed using the same method, albeit with the condition comprising 18% PEG 3350 and 0.1 M MES, pH 5.7. All crystals were cryo-protected with the mother liquor supplemented with 20% ethylene glycol prior flash-cooling in liquid nitrogen.

#### Structure Determination

Diffraction data for the ΔN90-PGAM5 and ΔN54-PGAM5 crystals were collected on in-house Rigaku FRE-Superbright and Diamond Light Source, respectively. Data were processed with MOSFLM (Powell et al., 2013) and subsequent scaled with SCALA (Evans, 2011). Initial apo structure was solved by SIRAS using the program SHELXD (Sheldrick, 2010) and SHARP (Bricogne et al., 2003). The improve phases obtained after solvent flattening were used for automated model building with Buccaneer (Cowtan, 2012). Structure solution of both phosphate complexes were achieved by molecular replacement with the program PHASER (McCoy et al., 2007) and the apo-structure as a model. Both PGAM5 structures were subjected to iterative cycles of manual building alternated with refinement using COOT (Debreczeni and Emsley, 2012) and REFMAC (Rinaldelli et al., 2014), respectively. TLS definitions were determined by the TLSMD server, and used in the final refinement step. Geometric correctness of both structures was verified by MOLPROBITY (Hintze et al., 2016). Data collection and refinement statistics are summarised in Table 1.

#### Analytical Ultracentrifugation

Sedimentation velocity experiments were performed at 4°C on a Beckman Optima XL-I Analytical Ultracentrifuge using an AnTi-50 rotor at the speed of 45,000 rpm. Proteins were prepared at a concentration of 50-60 μM in 50 mM Tris, pH 7.5, 300 mM NaCl and 0.5 mM TCEP. Radial absorbance at a wavelength of 280 nm were collected in continuous scan mode. Data were analyzed using the SEDFIT (Schuck, 2000) to calculate sedimentation coefficients (s) and differential sedimentation coefficient distributions (c(s) distributions), which were then normalized into the sedimentation coefficient in water at 20°C (*s°*_*20*, *W*_) by taking into account the solvent density (1.01378 g/ml) and viscosity (1.567x10^-2^ poise). Sedimentation Equilibrium (SE) experiments were performed at three protein concentrations (0.7, 0.46 and 0.35 mg/ml) in 50 mM HEPES, pH 7.5, 300 mM NaCl and 0.5 mM TCEP and two centrifugation speeds (12,000 rpm and 18,000 rpm) followed by a meniscus depletion run at 38,000 rpm. Samples were maintained at each speed for 22 hours before 5 scans were collected, followed by additional 2 hours and additional 5 scans. Data were evaluated with the software package Ultraspin using a self-association model followed by global analysis employing both speeds and plotted in Origin.

#### Immonoprecipitation Pull-Down Assays

HEK293 cells were transiently transfected with HA-tagged and flag-tagged PGAM5 constructs subcloned into pcDNA3 vector and cultured for 24 h. Cells were then lysed with the IP lysis buffer (50 mM Tris-HCl, pH 8.0, 150 mM NaCl, 1% deoxycholate, 1% Triton X-100, 10 mM EDTA, 1 mM phenylmethylsulfonyl fluoride, and 5 μg/mL aprotinin). Cell extracts were clarified by centrifugation, and the supernatants were immunoprecipitated with flag antibody gel (Anti-flag M2 affinity gel). The gels were washed 3 times with the IP lysis buffer. Cell extracts and immunoprecipitates were then resolved on SDS-PAGE and electroblotted onto polyvinylidene difluoride membranes. After blocking with 5% skim milk in TBS-T (50 mM Tris-HCl, pH 8.0, 150 mM NaCl, and 0.05% Tween 20), the membranes were probed with antibodies to flag (Anti-flag M2 affinity gel) and HA (Anti-HA high affinity) tags. The antibody-antigen complexes were detected using the ECL system.

#### Analytical Size Exclusion Chromatography

Three recombinant PGAM5 proteins, ΔN29-PGAM5, ΔN54-PGAM5 and ΔN90-PGAM5, were expressed and purified using the same protocol described for the preparation of proteins for crystallization, albeit without TEV cleavage. All size exclusion chromatography experiments were performed using Superdex Increase s200 10/300 GL (GE healthcare) in 50 mM Tris, pH 7.5 and 200 mM NaCl.

#### Hydrogen Exchange Mass Spectrometry

PGAM5 constructs ΔN29-PGAM5, ΔN54-PGAM5, ΔN61-PGAM5, ΔN90-PGAM5 were explored with continuous labelling hydrogen exchange experiments (time-course 10secs-2hrs). The proteins were diluted 10-fold into ^2^H_2_O, quenched, and labelled samples were digested online prior to chromatographic separation at 0°C. Electrospray MS analysis was carried out on a Waters QToF Premier coupled to a nanoACQUITY UPLC system modified for HDX applications (Wales et al., 2008). Deuterium incorporation with time was determined for all peptides of the different proteins and the results compared across all constructs.

### Data and Software Availability

Coordinates and structure factors of the structures reported in this study have been deposited to the PDB under accession codes 3MXO, 3O0T and 5MUF.

## Author Contributions

S.R.M. performed hydrogen-exchange mass spectrometry experiments and interpreted the data. S.P. purified proteins and cloned constructs. K.T., S.S., and S.K. performed IP experiments. P.F. performed and interpreted AUC experiments. M.S. performed analytical size-exclusion chromatography. A.C. performed crystallographic study. J.R.E., S.K., and H.I. supervised research. A.C. and S.K. wrote the paper with contributions from all authors.

## Supplemental Information

Document S1. Figures S1 and S2

Document S2. Article plus Supplemental Information
