# Supplementary material for: Structures of PGAM5 Provide Insight into Active Site Plasticity and Multimeric Assembly
Source: Structure. 2017 Jul 5;25(7):1089–1099.e3. doi: 10.1016/j.str.2017.05.020 (PMC5501728; doi:10.1016/j.str.2017.05.020)
Supplement: Document S1. Figures S1 and S2 [file mmc1.pdf]

Structure, Volume *25*

Supplemental Information

Structures of PGAM5 Provide Insight into Active Site Plasticity and Multimeric Assembly

Apirat Chaikuad, Panagis Filippakopoulos, Sean R. Marcsisin, Sarah Picaud, Martin Schröder, Shiori Sekine, Hidenori Ichijo, John R. Engen, Kohsuke Takeda, and Stefan Knapp

Structures of PGAM5 provide insight into active site plasticity and multimeric assembly

Apirat Chaikuad[1,2,6*], Panagis Filippakopoulos[2,3], Sean R. Marcsisin[4], Sarah Picaud[2,3], Martin Schröder[1], Shiori Sekine[6], Hidenori Ichijo[6], John R. Engen[4], Kohsuke Takeda[5], Stefan Knapp[1,2,7]

Supplementary information

Page 2 **Supplementary Figure S1. Selected top 20 similar structures to PGAM5 identified by DALI structural similarity search (extended from Figure 1C).**

Page 3 **Supplementary Figure S2. Analytical size exclusion chromatography (SEC) results (in relation to Figure 3E).**

```
PGAM5 SMDHYKAKATRHIFLIRHSQYHVDGSLEKDRTLTPLGREQAELTGLRLASLGLKFNKIVHSSMTRAIETTDIISRHLPGVCKVSTDLLREGAPVQYY
 4ij5 ---------MVKLILVRHAESEWVGRYQLDPDLSERGKKQAKLLAQELSRE--HLDVIYSSPLKRTYLTALEIAEAKN-LEVIKEDRIIEIDHGGES
 1h2f ---------ATTLYLTRHGETKWERRMQQDSPLTEKGRQDAMRLGKRLEA--VELAAIYTSTSGRALETAEIVRGG-RLIPIYQDERLREIHLRGER
 1ujb ----------MQVFIMRHGDAALDAASDSVRPLTTNGCDESRLMANWLKGQKVEIERVLVSPFLRAEQTLEEVGDCLNLPSAEVLPELTPC------
 3eoz -------NTTKHIILVRHG--------------TKEGCKQADITGKKLKDINKKVSVIYHSDXIRAKETANIISKYFPDANLINDPNLNEGT-----
 3f2i ----------XELYLIRHGIAEAQ-IKDEERELTQEGKQKTEKVAYRLVKLGRQFDLIVTSPLIRARQTAEILLASG--CQLEESNHLAPN------
 3e9c -------MLTFALTIVRHGET--------DTPLSDTGHQQAAAAGRYLKD--LHFTNVFVSNLQRAIQTAEIILGNNSATEMILDPLLRERGF---G
 4qih ---------ARRLVMLRHGQTDYGSRMQLDTELSELGRTQAVAAAEVLGK--RQPLLIVSSDLRRAYDTAVKLGERTG-LVVRVDTRLRETHLHGGE
 3r7a -----TDSNVVTLYVTRHGKTILNHRAQ--SPLVEKGVEVATNLGTGLKD--IHFXNAYSSDSGRAIETANLVLKYQSKLKLEQRKKLRELNF-DWE
 2a6p --------RNHRLLLLRHGETAW----GTEVELTDTGRTQAELAGQLLGELELDDPIVICSPRRRTLDTAKLAGL----TVNEVTGLLAEWDYAGES
 3hjg --------KTLNIYLMRHGKVD-AAPG--DLKVK---EAEQQQIAMAWKTKGYDVAGIISSPLSRCHDLAQILAEQQL-LPMTTEDDLQEMDFNAES
 3d6a --------SRRGILVIRHGERVD-FEND--PPLSSCGIFQARLAGEALLDSGVRVTAVFASPALRCVQTAKHILEELKKLKIRVEPGIFEWMKPAES
 5ak0 -KKLT-HVQPRTIYLCRHGENEHQGRIGGDSGLSSRGKKFASALSKFVEEQNLKDLRVWTSQLKSTIQTAEALR-----LPYEQWKALNEIDATGES
 1c80 ---------MRSIYLCRHGESELRGRIGGDSGLSARGKQYAYALANFIRSQGISSLKVWTSHMKRTIQTAEALG-----VPYEQWKALNEIDAKGES
 1xq9 -------MTTYTLVLLRHGESTWENKFTTDVPLSEKGEEEAIAAGKYLKEKNFKFDVVYTSVLKRAICTAWNVLKTLLHVPVVKTWRLNERHYFTEC
 4gpz ---------AYKLVLIRXGESAWENRFSYDADLSPAGHEEAKRGGQALRDAGYEFDICFTSVQKRAIRTLWTVLDAQMWLPVVRTWRLNERHYSCES
 5hr5 -KLTR-HVHPRTIYLCRXGESEFLGKIGGDSGLSVRGKQFAQALRKFLEEQEIADLKVWTSQLKRTIQTAESLG-----VTYEQWKILNEIDAGGES
 2ikq ---------KRCLFVCRHGERMDVYEKD--APITVFGCMQARLVGEALLESNTVIDHVYCSPSLRCVQTAHNILKGLQHLKIRVEPGLFEWTKISES
 2axn -KLTR-HVQPRTIYLCRHGENEHQGRIGGDSGLSSRGKKFASALSKFVEEQNLKDLRVWTSQLKSTIQTAEALR-----LPYEQWKALNEIDATGES
 5htk -KLTR-HVQPRTIYLCRXGESEFLGKIGGDSGLSVRGKQFAQALRKFLEEQEITDLKVWTSQLKRTIQTAESLG-----VPYEQWKILNEIDAGGES
 4emb ---------XYKLVLVRHGESEWENLFTTDVKLSDKGIDEAVEAGLLLKQEGYSFDIAFSSLLSRANDTLNIILRELGYISVKKTWRLNERHYSTEC

PGAM5 EDGARIEAAFRNYIHRADARQEEDSYEIFICHANVIRYIVCRALQFPPEGWLRLSLNNGSITHLVIRPNGRVALRTLGDTGFMPPDKITRS
 4ij5 -LASVYNRVKG-FLEEKRHW---NQTVVVVSHTVPMRAMYCALLGVDLSKFWSFGCDNASYSVIHMEER-RNVILKLNITCHLG-EFYVEA
 1h2f -FCDVQQRALEAVQIVDR----EGETVLIVTHGVVLKTLMAAFKDTPLDHLWSPYMYGTSVTIIEVDG-GTFHVAVEGDVSHIE--EVKEV
 1ujb ----DVGLVSAY-LQALTNEG--VASVLVISHLPLVGYLVAELCPGET-----PMFTTSAIASVTLDESGNGTFNWQ-MSPCNLK------
 3eoz ---KRINKAYETYF-YKPSG-DEDEYQLVICHGNVIRYFLCRALQIPWLRFSSYNC---GITWLVLDDEGSVVLRE--VSHL---------
 3f2i ----NIFNWLDYWLKPKN--FPENAQIAIVGHEPCLSNWTEILLWGEAK--DSLVLKKAGXIGLKLPEIGRSQXFWLTPPYLLL-------
 3e9c ETLEQVKTRFKMFLKSMFEEHGSPVHALMVSHGAFIRISVRHLVED------FSPCPNTGISRFIFTIATRIQGVFINRKDHL--------
 4qih SRVDVAARSRP-LVAEVASEPDEPDPVVLVAHGGLIAALSAALLKLNWPAL--GGMGNASWTQLSGHWSIRWRLDVWNAS-----------
 3r7a LFSTRIKAEIDKISEEAKDG---GGNVLVVVHGLLITTLIEXLD---SSKTK-LGVENASVTKIVYQD-GIYTVESVGDXSYVA-------
 2a6p QVNDRADSAVA-LALEH-----SSRDVLFVSHGHFSRAVITRWVQLPLAEGSRFAMPTASIGICGFE-HGVRQLAVLGLTGH---------
 3hjg TFSQRVSRAWSQIINDIN------DNLLIVTHGGVIRIILAHVLGVDWPQWYTLAIGNASVTHITITIDIYASVRSIGVPLVE--------
 3d6a QYVERCAVSMGQI-INTC--PQDMGITLIVSHSSALDSCTRPLLGLPPRDFAQLKIPSLGMCFCEENRDGKWDLVN----KTLTHG-----
 5ak0 DLVQRLEPVIME-LERQ-------ENVLVICHQAVLRCLLAYFLDKSAEEMPYLKCPLHTVLKLTPV-AYGCRVES--IYLNV---ESVCT
 1c80 DLVQRLEPVIME-LERQ-------ENVLVICHQAVMRCLLAYFLDKSSDELPYLKCPLHTVLKLTPV-AYGCRVES--IYLNV--------
 1xq9 DTVERVLPFWFDHIAPD-----ANKKVMVAAHGNSLRGLVKHLDNLSEADVLELNIPTGVPLVYELDENLKPIKHY--YLLDELKKKMD--
 4gpz DTIARALPFWNEEIVPQ-----EGKRVLIAAHGNSLRGIVKHLEGLSEEAIMELNLPTGIPIVYELDKNLKPKPMQ--FLGDTVRK-----
 5hr5 DLVQRLEPVIME-LERQ-------GNVLVISHQAVMRCLLAYFLDKGADELPYLRCPLHTIFKLTPV-AYGCKVET--IKLNV---EAVNT
 2ikq TYINRSFQVTKEIISECKSKG---NNILIVAHASSLEACTCQLQGLSPQDFVQMKIPYLGFCSCEELETGIWQLTD----LPLTHG-----
 2axn DLVQRLEPVIME-LERQ-------ENVLVICHQAVLRCLLAYFLDKSAEEMPYLKCPLHTVLKLTPV-AYGCRVES--IYLNV---ESVCT
 5htk DLVQRLEPVIME-LERQ-------GNVLVISHQAVMRCLLAYFLDKGADELPYLRCPLHTIFKLTPV-AYGCKVET--IKLNV---EAVNT
 4emb DTVARVIPYWTDEIAKE-----EGKKVIVAAHGNSLRALVKYFDNLSEEDVLKLNIPTGIPLVYELDKDLNPIKHY--YLGDESK-KKAX-
```

PDB ID	Organism	Protein name	Dali Z score	rmsd (Å)	Sequence identity (%)
4ij5	*Hydrogenobacter thermophilus*	Phosphoserine Phosphatase	20.4	2.6	16
1h2f	*Geobacillus stearothermophilus*	PHOSPHATASE yhfR	19.8	2.5	15
1ujb	*Escherichia coli*	histidine phosphatase SixA	18.9	2.3	14
3eoz	*Plasmodium falciparum*	Phosphoglycerate mutase (*Pf*PGM2)	18.7	2.1	40
3f2i	*Nostoc sp. pcc 7120*	Alr0221 protein	18.7	2.3	21
3e9c	*Danio rerio*	Zgc:56074	18.0	2.2	21
4qih	*Mycobacterium tuberculosis*	Glucosyl-3-phosphoglycerate phosphatase	18.0	2.9	19
3r7a	*Bacillus anthracis*	Phosphoglycerate mutase, putative	17.2	3.0	20
2a6p	*Mycobacterium tuberculosis*	PHOSPHOGLYCERATE MUTASE GPM2, MT3310	17.0	2.6	25
3hjg	*Vibrio parahaemolyticus*	Putative alpha-ribazole-5'-phosphate phosphatase CobC	16.6	2.7	20
3d6a	*Mus musculus*	Sts-2	16.3	2.4	16
5ak0	*Homo sapiens*	6-PHOSPHOFRUCTO-2-KINASE/FRUCTOSE-2,6-BISPHOSPHATASE 3	16.3	3.1	17
1c80	*Rattus norvegicus*	FRUCTOSE-2,6-BISPHOSPHATASE	16.1	3.0	21
1xq9	*Plasmodium falciparum*	phosphoglycerate mutase	15.9	3.2	20
4gpz	*Homo sapiens*	Phosphoglycerate mutase 1	15.9	3.0	22
5hr5	*Bos taurus*	6-phosphofructo-2-kinase/fructose-2,6-bisphosphatase 2	15.9	3.1	18
2ikq	*Mus musculus*	Sts-1	15.9	2.5	16
2axn	*Homo sapiens*	6-phosphofructo-2-kinase/fructose-2,6-biphosphatase 3 (6PF-2-K/Fru- 2,6-P2ASE brain/placenta-type isozyme) (iPFK-2)	15.8	3.1	17
5htk	*Homo sapiens*	6-phosphofructo-2-kinase/fructose-2,6-bisphosphatase 2	15.8	3.2	18
4emb	*Borreliella burgdorferi*	2,3-bisphosphoglycerate-dependent phosphoglycerate mutase	15.8	3.1	22

Supplementary Figure S1. Selected top 20 similar structures to PGAM5 identified by DALI structural similarity search extended from Figure 1C. Shown are the structure-based sequence alignment, and the DALI statistics.

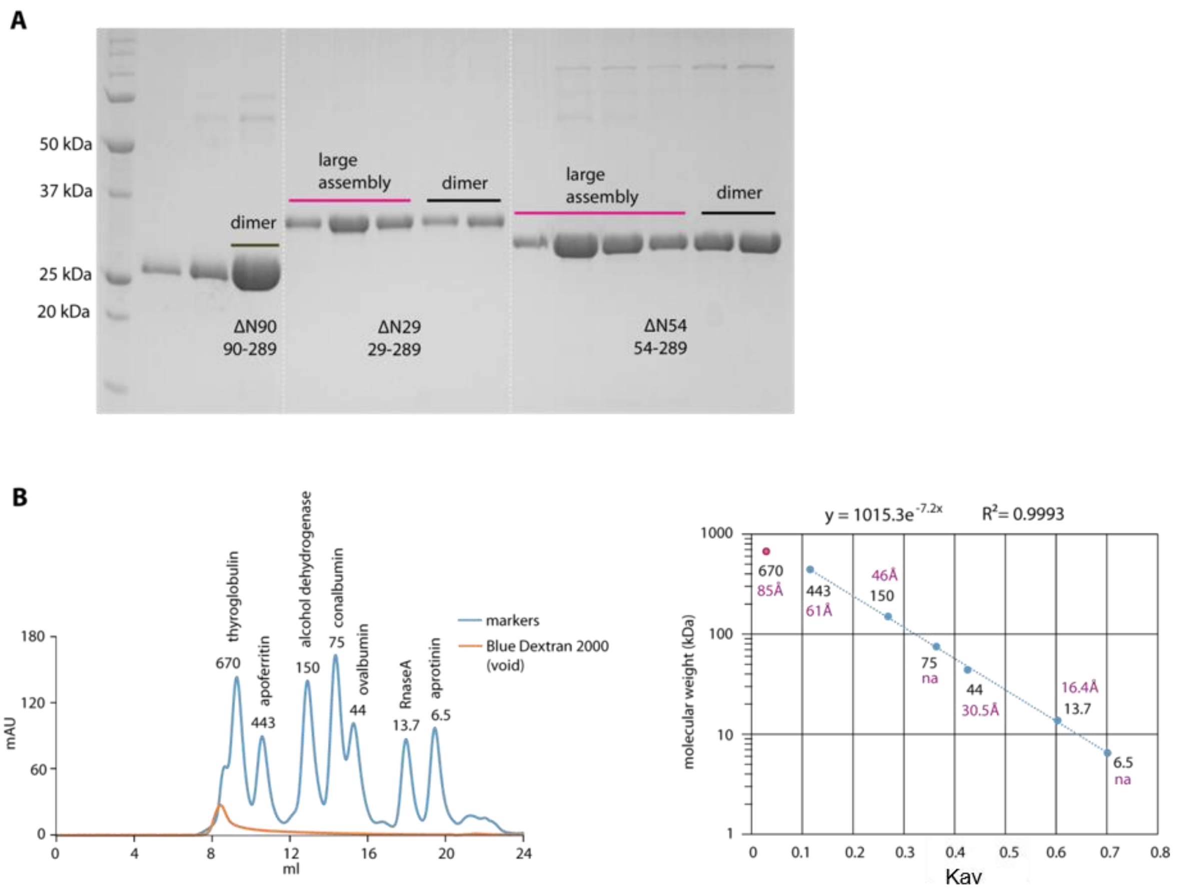

Supplementary Figure S2. Analytical size exclusion chromatography (SEC) results (in relation to Figure 3E). A: SDS-PAGE analyses of the proteins in the peak elution fractions in relation to the chromatogram in Figure 3E. **B:** the chromatogram of the protein markers (left) and the calibration curve (right). The values in black indicated the molecular weights of each protein marker in kDa, and the numbers in magenta in the right panel indicated the estimated globular radii. Kav values were calculated from the following equation: Kav = (Ve-Vo)/(Vt-Vo), where Ve = elution volume, Vo = void volume and Vt = column volume.
